# Protective role of trametenolic acid B against sevoflurane-induced cognitive impairments by its different regulatory modalities of mir-329-3p in neurons and microglia

**DOI:** 10.1186/s10020-022-00477-6

**Published:** 2022-07-03

**Authors:** Jun Chen, Shuo Feng, Linyan Li, Shujie Qiu, Yanwu Jin, Yingui Sun

**Affiliations:** 1 Department of Anesthesiology, Weifang Medical University, Weifang, 261053 Shandong China; 2 Department of Gynecology, Affiliated Hospital of Weifang Medical University, Weifang, 261031 Shandong China; 3 Department of Anesthesiology, Affiliated Hospital of Weifang Medical University, No. 7166 Baotong West Street, Weifang, 261031 Shandong China; 4 Department of Anesthesiology, The Second Hospital, Cheeloo College of Medicine, Shandong University, Jinan, 250033 Shandong China

**Keywords:** Postoperative cognitive dysfunction, Trametenolic acid B, Sevoflurane, Neurotoxicity, Neuroinflammation, MicroRNAs

## Abstract

**Background:**

Postoperative cognitive dysfunction induced by anesthetics commonly occurs in elderly patients. This study aimed to evaluate the protective role of trametenolic acid B (TAB) in sevoflurane-induced cognitive impairments, and explore the underlying mechanisms.

**Methods:**

Animal and cell experiments were performed in rats, differentiated PC12 and HAPI cells by exposing to 2% sevoflurane for 5 h. Different concentration (20, 40 and 80 µg/mL) of TAB was administrated in rats and cells. The cognitive function of rats was evaluated using the Morris water maze test and fear conditioning test. The cell proliferation and apoptosis were investigated using a CCK-8 assay and the flow cytometry. Pro-inflammatory cytokines in microglia were measured using ELISA kits. A miRNA microarray assay was conducted to screen differentially expressed miRNAs by TAB in both PC12 and HAPI cells. The luciferase reporter assay and western blot assay were used to assess the E2F1/CCNA2 and NF-κB pathways.

**Results:**

TAB significantly alleviated sevoflurane-induced cognitive impairments in rats, improved PC12 cell viability, and inhibited the neuroinflammation of HAPI cells. miR-329-3p was downregulated in PC12 cells but upregulated in HAPI cells by TAB treatment, which mediated the effects of TAB on neurotoxicity and neuroinflammation. E2F1 and NF-κB P65 were two targets of miR-329-3p, and the E2F1/CCNA2 and NF-κB pathways were inhibited by miR-329-3p in PC12 and HAPI cells, respectively.

**Conclusions:**

All the results provide evidence for the protective role of TAB against sevoflurane-induced cognitive impairments, which was achieved by alleviating neurotoxicity and neuroinflammation through differentially regulating miR-329-3p in neurons and microglia.

## Introduction

Postoperative cognitive dysfunction (POCD) is a commonly occurred postoperative complication in central nervous system especially for elderly patients, which is characterized by cognitive impairments after anesthesia and surgery (Lin et al. 2020). Anesthesia and aging have been determined as two major risk factors of POCD, and patients received inhalation anesthesia exhibit high POCD rate compared with those with intravenous anesthesia (Miller et al. 2018). Thus, sevoflurane, as the most widely used inhalation anesthetic, has attracted increasing attention for the cognitive dysfunction it causes (Guo et al. 2020). Neurotoxicity and neuroinflammation induced by sevoflurane are key events for the aggravation of POCD, making them become the breakthrough points to alleviate POCD (Mutch et al. 2018; Safavynia and Goldstein 2018).

Traditional Chinese Medicine (TCM) active ingredients have attracted increasing attention on their critical biological effects on the progression of various diseases (Dai et al. 2021; Wei et al. 2021; Wang et al. 2021). Some of the monomers of TCM have important neuroprotective effects, such as ginsenoside Rg1 (Yang et al. 2020), chikusetsu saponin V (Zhang et al. 2021) and mangiferin (Peng et al. 2019). For the cognitive impairments induced by sevoflurane, chikusetsu saponin IVa (Shao et al. 2020) ampelopsin (Liu et al. 2020a, b) and cistanches (Peng et al. 2020) have been demonstrated to exert beneficial effects on cognitive function by reducing neuroinflammation or improve neuronal cell viability. Trametenolic acid B (3β-hydroxylanosta-8, 24-diene-21-oic acid, TAB) is a kind of lanostane triterpenoid and isolated from the *trametes lactinea* Pat. TAB has been reported to exert various pharmacological activities, including anti-cancer and anti-microbial effects (Zhang et al. 2013; Leliebre-Lara et al. 2016). A previous study has found that TAB could protect against cerebral ischemia and reperfusion injury by improving neural cell viability through the microRNA-10a (miR-10a)/PI3K/AKT/mTOR pathway (Wang et al. 2019), which indicated the potential relationship between TAB and central nerve system disorders. However, it remains unknown whether TAB also has protective effects in sevoflurane-induced cognitive impairments.

MicroRNAs (miRNAs) are a group of small non-coding RNAs with important biological function in various human diseases (Xia et al. 2019). They can directly bind to the 3’-untranslated region (UTR) of target genes, leading to translation inhibition or mRNA degradation and ultimately inhibit the expression of target genes (Sun et al. 2018). In the pharmacological activities of TCM, some miRNAs also play pivotal roles. For example, tanshinone IIA could inhibit acute myeloid leukemia cell viability by upregulating miR-497-5p (Nie et al. 2020). miR-144 expression was inhibited by ginsenoside Rg1 in ischemic/reperfusion-induced neuronal injury cell models, which contributed to neuronal viability (Chu et al. 2019). According to a miRNA microarray analysis, this study found that miR-329-3p was differentially expressed in sevoflurane-treated neurons and microglia after TAB administration. Interestingly, the expression patterns of miR-329-3p induced by TAB in neurons and microglia were opposite. miR-329-3p is a cell viability- and inflammation-related miRNA. It could target E2F transcription factor 1 (E2F1), which is an important cell cycle- and proliferation-related molecular, and subsequently leads to the inhibition in cell viability in various kinds of cell lines (Lin et al. 2019). In addition, miR-329-3p has been documented to suppress placental inflammation by directly inhibiting P65, which is a key element of the nuclear factor-κB (NF-κB) signaling (Garg et al. 2013). However, the understanding of the specific role of miR-329-3p is limited in sevoflurane-induced cognitive impairments.

Therefore, in this study, the effects of TAB on cognitive impairments induced by sevoflurane were investigated in aged rats. In addition, the changes in neuronal viability and the miR-329-3p/E2F1/cyclin A2 (CCNA2) pathway led by TAB treatment were assessed in PC12 cells. In HAPI microglia, this study examined the effects of TAB on neuroinflammation and related molecular mechanism involved in the miR-329-3p/NF-κB pathway. The results of this study demonstrate that TAB may be a promising therapeutic agent for sevoflurane-induced POCD. The neuronal viability and neuroinflammation changes and the expression in miR-329-3p/E2F1 axis and miR-329-3p/NF-κB P65 axis may explain the neuroprotective mechanisms of TAB in sevoflurane-induced POCD.

## Materials and methods

### Materials and reagents

RPMI-1640 and Dulbecco’s Modified Eagle’s Medium (DMEM) culture medium, fetal bovine serum (FBS), trypsin, penicillin, streptomycin, dimethyl sulfoxide (DMSO) and phosphate buffered saline (PBS) were purchased from Gibco (Carlsbad, CA, USA). miR-329-3p mimic, miR-329-3p inhibitor, mimic negative control (NC) and inhibitor NC were synthesized in GenePharma (Shanghai, China). Lipofectamine 3000, TRIzol isolation kit and cDNA synthesis kit were purchased from Invitrogen (Carlsbad, CA, USA). The Cell Counting Kit-8 (CCK-8) was purchased from Sigma (St. Louis, MO, USA). The Annexin V-FITC Apoptosis Detection kit was obtained from BD Pharmingen (San Diego, CA, USA). Enzyme-linked immunosorbent assay (ELISA) kits were purchased from R & D System (Minneapolis, USA). The miRNeasy Mini kit was purchased from Qiagen (Hilden, Germany), and miRNA Complete Labeling and Hybridization kit and miRNA Microarray Release were purchased from Agilent (Santa Clara, CA, USA). The SYBR premix Ex Taq II kit was purchased from TaKaRa (Dalian, China) Primary antibodies and secondary antibodies for western blotting were purchased from Cell Signaling Technology (Danvers, MA, USA). Western ECL detection reagent was purchased from Beyotime Biotechnology (Shanghai, China). TAB (purity > 98%) was dissolved by DMSO at high concentration, which was prepared and stored at −20 ℃ for further experiments.

### Animals and sevoflurane exposure

Animal experiments used Sprague-Dawley (SD) rats (20-month old, male), which were purchased from Shanghai SLAC Laboratory Animal Co., Ltd (Shanghai, China) and kept in a standard animal housing conditions: temperature of 22 ± 2℃, humidity of 60 ± 5%, 12:12 h light/dark cycle, and free access to water and food. After 7 days acclimatization, the rats were divided into four groups (18 rats in each group): (Lin et al. 2020) normal group (Normal), the rats in this group inhaled normal air for 5 h; (Miller et al. 2018) SEV group, rats inhaled 2% sevoflurane for 5 h, and 70% O2 was used as the carrier with a gas flow rate of 1.5 L/min; (Guo et al. 2020) SEV + vehicle group, rats received normal saline (0.1 mL/100 g) before sevoflurane exposure; (Mutch et al. 2018) SEV + TAB-L group, rats were treated with 20 mg/kg TAB orally once a day for 7 consecutive days before sevoflurane exposure; (Safavynia and Goldstein 2018) SEV + TAB-M group, 40 mg/kg TAB wad administrated for 7 days; (Dai et al. 2021) SEV + TAB-H group, 80 mg/kg TAB was used for 7 days. The rats in each group were assigned to the cognitive and behavioral tests after blood collection from the internal jugular vein. Serum samples were isolated from the blood by centrifugation and used for subsequent analyses. All the animal experimental procedures were approved by the Experimental Animal Care and Use Committee of Weifang Medical University (Ethical Approval number: A-0253).

### Cell culture and treatment

Rat neuronal differentiated PC12 cells and rat microglia HAPI cells were purchased from the Cell Bank of Type Culture Collection of Chinese Academy of Science (Shanghai, China). PC12 cells were cultured using RPMI-1640 medium and HAPI cells were kept at DMEM medium in a humidified atmosphere with 5% CO_2_ at 37℃. In the culture medium, 10% FBS and 1% penicillin-streptomycin solution were added.

Cells were cultured at an atmosphere with 5% CO_2_ or 2% sevoflurane for 5 h to establish cell anesthesia models. For TAB administration, cells were treated with low (20 µg/mL), moderate (40 µg/mL) or high (80 µg/mL) levels of TAB at 48 h before sevoflurane exposure. At 48 h-preanesthetic treatment, cells were seeded into 6-well plates, and cell transfection was performed after cells reached 80% confluence using Lipofectamine 3000 following manufacture’s protocols. Following were the sequence information of the transfection vectors (from 5’ to 3’): miR-329-3p mimic, AACACACCCAGCUAACCUUUUU; miR-329-3p inhibitor, AAAAAGGUUAGCUGGGUGUGUU; mimic NC, UUCUCCGAACGUGUCACGU; inhibitor NC, CAGUACUUUUGUGUAGUACAA.

### Cognitive and behavioral tests

Morris water maze (MWM) test was used to evaluate the cognitive function of rats after the indicated treatment of each group. The MWM had a pool with a depth of 50 cm and a diameter of 120 cm, which contained water (25 ± 1℃) and a hidden platform. Four quadrants were divided in the pool, including quadrant I, II, III and IV. The platform was placed in the III quadrant and 1 cm below the water surface. This test had two major parts: acquisition training and probe trial. The rats were firstly trained for 5 consecutive days, during which the rats were placed at random positions and allowed to find the platform within 60 s and stay on the platform for 10 s. If the rats did not find the platform within 60 s, they were manually placed on the platform for 10 s. The escape latency and speed were recorded. After 5 days training, the platform was removed from the pool, and rats were placed in the quadrant I for swimming for 60 s. The number of crossings of the platform, the time spent in the third quadrant and the swimming trajectory were recorded.

Fear conditioning test (FCT) was used to evaluate the spatial memory and learning abilities of rats. Rats were placed in the chamber for FCT on 1 day after anesthesia to adapt the chamber for 120 s, then were exposed with a sound stimulus (20 s, 80 dB). After 25 s of interval, the rats received an electric foot shock (2 s, 0.75 mA). The sound stimulus and foot shock were repeated for 3 times with 1 min of interval. Two days after the sound stimulus and foot shock, fear conditioning memory was evaluated. Rats were put in the same chamber without sound stimulus or shock, and the percentage of freezing time was recorded.

Open field test (OFT) was performed to evaluate the behavior of rats. After 1 day of anesthesia, the rats were placed in a chamber (50 cm × 50 cm × 40 cm; inner walls were white color) used for the OFT. A rat was placed in the center of the chamber for 5 min. The whole distance of the trajectory and the stay time at the central area of rats were recorded.

The same rats were not used repeatedly in the above 3 tests.

### Cell proliferation assay

The cell proliferation of PC12 was determined using the CCK-8. Cells with a density of 5 × 10^3^ cells per well were seeded into 96-well plates and cultured at 37℃ in the incubators with 5% CO_2_ for 2 h for adherence. Then, 10 µL CCK-8 reagent was added into each well with further 2 h incubation. The absorbance of the incubate was measured at a wavelength of 450 nm. Each experiment was repeated for three times.

### Cell apoptosis evaluation

PC12 cells were collected after the indicated treatment and washed using phosphate-buffered saline (PBS) twice and centrifuged for 10 min at 1000 r/min. An Annexin V-FITC Apoptosis Detection Kit was used to investigate cell apoptosis. After cell collection, cells were stained with Annexin V-FITC and PI at room temperature for 15 min under a dark condition, and the cell apoptosis rate was examined using a FACS Calibur Flow Cytometer (Becton, Dickinson and Company, CA, USA). Each experiment was repeated for three times.

### Evaluation of neuroinflammation in microglia

Neuroinflammation was evaluated by examining the levels of pro-inflammatory cytokines in the supernatant of microglia HAPI cells. The enzyme-linked immunosorbent assay (ELISA) kits were adopted to measure the concentration of interleukin (IL)-1β, IL-6 and tumor necrosis factor (TNF)-α following the manufacturers’ instructions.

### RNA extraction and miRNA microarray assay

Sevoflurane-treated PC12 and HAPI cells administrated with or without Table (40 µg/mL) were collected, and the total RNA, including miRNAs, was extracted using a miRNeasy Mini Kit. The RNA samples were pooled to perform miRNA microarray analysis, which were firstly labeled by the Agilent miRNA Complete Labeling and Hybridization Kit as per the manufacturer’s instructions, then were hybridized to the Agilent miRNA Microarray Release at 65℃ for 20 h. The results were scanned on an Agilent DNA Microarray Scanner with the Scan Control software (Agilent Technologies), and the results were imported into GenePix Pro 6.0 software for data extraction.

### Quantitative real time PCR (qRT-PCR)

Total RNA from PC12 and HAPI cells was collected using TRIzol reagent, then was reversely transcribed into cDNA using a cDNA synthesis kit. The SYBR premix Ex Taq II kit was used to perform qPCR on an ABI 7900HT instrument (ABI, NY, USA), and the reaction conditions were as follows: 94℃ 10 min followed by 40 cycles of 94℃ for 30 s, 58℃ for 20 s, 72℃ for 30 s. The sequences of primers were listed in Table 1. The relative expression values were calculated using the 2^−△△Ct^ method and normalized to U6 (internal control for miR-329-3p) or GAPHD (internal control for E2F1 and P65).

**Table 1:** Sequences of primers

Category	Sequences (5’–3’)	
miR–329–3p	Forward	GCCGAGAACACACCCAGCT
	Reverse	CTCAACTGGTGTCGTGGA
E2F1	Forward	CCGTGGACTCTTCGGAGAAC
	Reverse	ATCCCACCTACGGTCTCCTC
NF–κB P65	Forward	GAAGGGAGGGAGTTTGGCTC
	Reverse	GTGGATCCTTGGTGACCAGG
U6	Forward	CTCGCTTCGGCAGCACAT
	Reverse	AACGCTTCACGAATTTGCGT
GAPDH	Forward	TGAAGGGTGGAGCCAAAAG
	Reverse	AGTCTTCTGGGTGGCAGTGAT

### Dual-luciferase reporter assay

PC12 and HAPI cells were seeded and cultured in 24-well plates, then were co-transfected with miR-329-3p mimic or mimic NC with the wild type (WT) or mutant type (MUT) 3’-UTR of E2F1 or P65 using Lipofectamine 3000 following the manufacture’s protocols. After 24 h of incubation, the luciferase activity of the cells was detected by a Dual Luciferase Reporter Assay Program (Promega, WI, USA) and normalized to the *Renilla* luciferase signal.

### Western blot assay

Total protein was extracted from PC12 and HAPI cells using RIPA lysis buffer (Beyotime, Shanghai, China), than was separated by 10% SDS-PAGE. The proteins were transferred onto polyvinylidene fluoride (PVDF) membranes (Millipore, Billerica, MA, USA), and were blocked with 5% skim milk. The membranes were firstly incubated with primary antibodies, including rabbit anti-E2F1 (1:1000), anti-CCNA2 (1:1000), anti-P65 (1:1000) and anti-GAPDH (1:1000), at 4℃ overnight, then were incubated with HRP-labelled goat anti-rabbit secondary antibody (1:1000) for 2 h at 37℃. After the incubation, bands were visualized by an ECL system (Millipore, Billerica, MA, USA).

### Statistical analysis

The data were expressed using mean ± SD and analyzed using SPSS 26.0 statistical software (SPSS Inc., Chicago, IL) and GraphPad Prism 7.0 software (GraphPad Software, Inc., USA). The differences between groups were compared using Student’s t test or one-way ANOVA followed by Tukey’s test. The differences were considered statistically significant when *P* value was less than 0.05.

## Results

### TAB prevents sevoflurane-induced cognitive impairment in aged rats

**Fig. 1:**
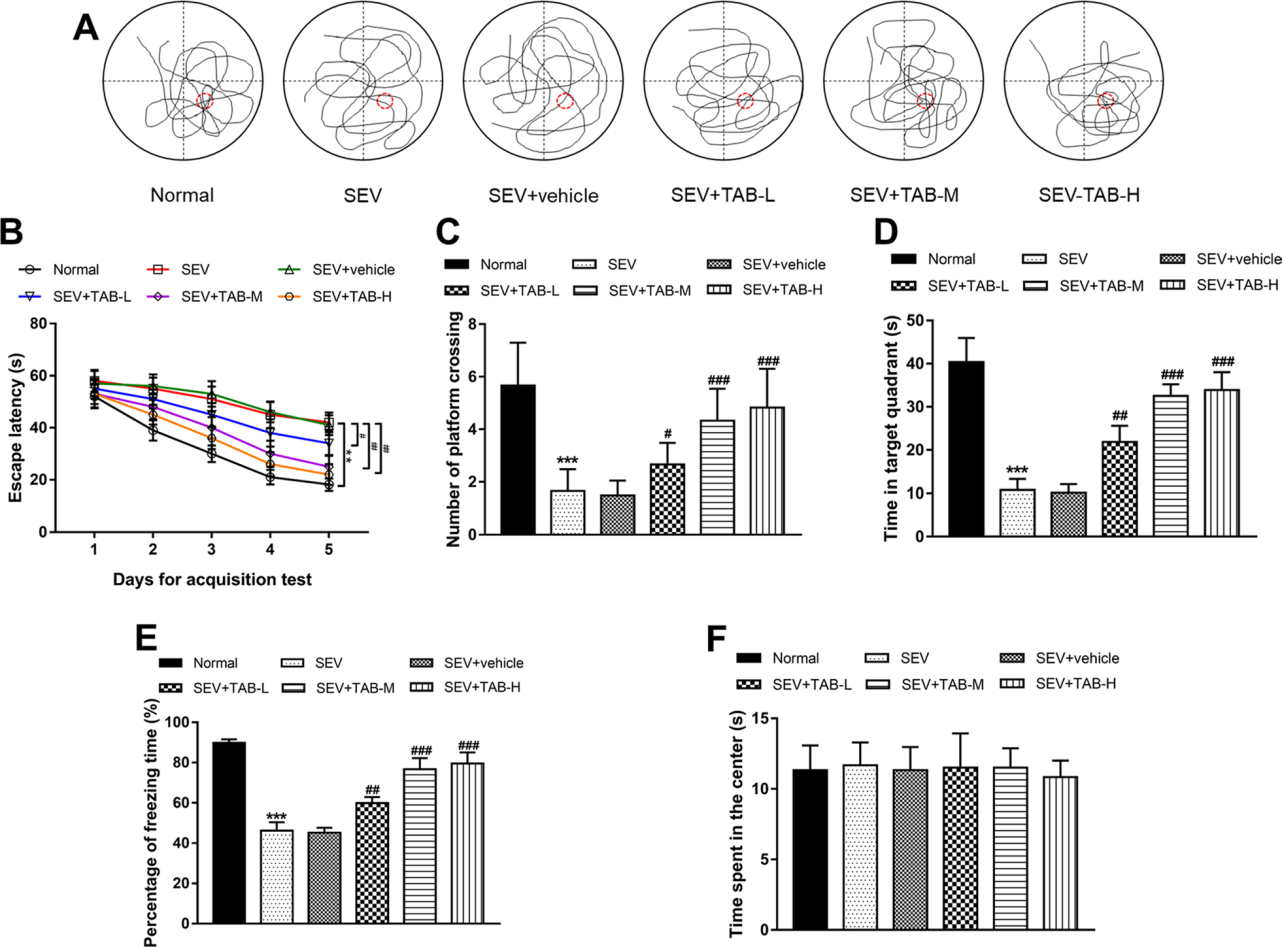
TAB alleviated the cognitive function of rats exposed to sevoflurane. **A** The representative trajectories of the rats in the probe trial from the MWM test. **B** Sevoflurane inhalation led to longer escape latency time, while TAB reduced this time in rats. **B** TAB administration increased the number of platform crossing, which was reduced by sevoflurane. **C** The time in target quadrant of rats was shorter in sevoflurane inhalation group than the normal group, but it was promoted by TAB administration. **E** The freezing time in FCT test was reduced in rats exposed to sevoflurane, and this effect was reversed by TAB treatment. **F** Time spent in the center of PFT test revealed that sevoflurane and TAB had no significant effects on the behavior of rats. **P < 0.01, ***P < 0.001 compared to normal group; ^#^*P* < 0.05, ^##^*P* < 0.01, ^###^*P* < 0.001 compared to SEV group

The MWM test was used to evaluate the cognitive function of rats, and the representative trajectories of the rats in the probe trial could be observed in Fig. 1A. As shown in Fig. 1B, during the training period, sevoflurane significantly increased the escape latency of rats (*P* < 0.01). The number of platform crossing and time in target quadrant were reduced in rats exposed to sevoflurane (Fig. 1C, D). In the rats administrated with TAB, the escape latency was decreased and the platform crossing number and time in target quadrant were elevated compared with those treated only sevoflurane exposure (all P < 0.05), and these effects showed dose dependence, which evidenced by the significant changes in TAB-M (40 µg/mL) and TAB-H (80 µg/mL) (both *P* < 0.01, Fig. 1A–D). From the results of FCT test, the percentage of freezing time was significantly shorter in rats inhaled sevoflurane than those in normal group (*P* < 0.001), while the sevoflurane-induced decreased freezing time was increased by TAB treatment with the significant changes in TAB-M and –H groups (both *P* < 0.001, Fig. 1E). To examine the behavior changes of rats, an OFT test was performed, and no significant changes were observed in the time spent in the center of rats (all *P* > 0.05, Fig. 1F).

### TAB promotes cell viability and inhibits cell apoptosis in sevoflurane-treated PC12 cells

**Fig. 2:**
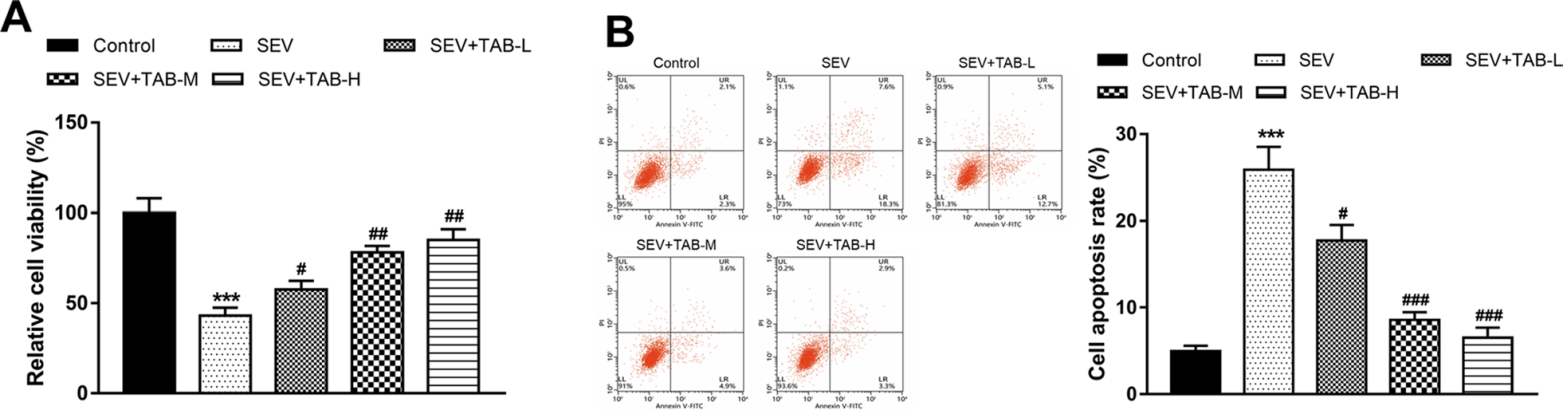
Effects of TAB on PC12 cell viability and apoptosis. **A** PC12 cell viability was inhibited by sevoflurane, but this change was reversed by TAB treatment. **B** Sevoflurane promoted PC12 cell apoptosis, but TAB could significantly reduce the increased cell apoptosis rate induced by sevoflurane. ***P < 0.001 compared to normal group; ^#^*P* < 0.05, ^##^*P* < 0.01, ^###^*P* < 0.001 compared to SEV group

By sevoflurane exposure, the cell viability of PC12 was significantly inhibited, but the cell apoptosis rate of PC12 was significantly increased compared with the control cells (both *P* < 0.001, Fig. 2). In the cells incubated with TAB, it is found that the sevoflurane-induced inhibition in cell viability and increase in cell apoptosis were all released, which manifested by the increased cell viability and decreased cell apoptosis (all *P* < 0.05). In addition, similar with the results in rats, the medium and high dose of TAB led to more significant results in cell viability and apoptosis in sevoflurane-treated PC12 cells (both *P* < 0.01 for cell viability, and both *P* < 0.001 for cell apoptosis).

### Inflammatory responses are alleviated by TAB in HAPI cells exposed to sevoflurane

**Fig. 3:**
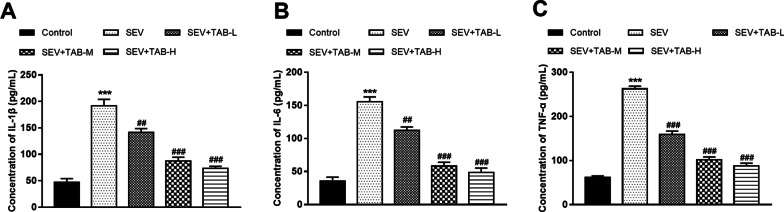
Effects on TAB on the inflammatory responses of HAPI cells. The concentrations of IL-1β (**A**), IL-6 (**B**) and TNF-α (**C**) were markedly upregulated by sevoflurane, but were significant inhibited by TAB treatment. ***P < 0.001 compared to normal group; ^##^*P* < 0.01, ^###^*P* < 0.001 compared to SEV group

Microglia HAPI cells showed significantly activated inflammatory responses by sevoflurane exposure, which can be demonstrated by the markedly increased levels of IL-1β, IL-6 and TNF-α (all *P* < 0.001, Fig. 3). By TAB administration, we observed that the upregulated pro-inflammatory cytokine levels were significantly reduced (all *P* < 0.01).

### TAB treatment leads to the differentially expressed mir-329-3p in sevoflurane-treated PC12 and HAPI cells

**Fig. 4:**
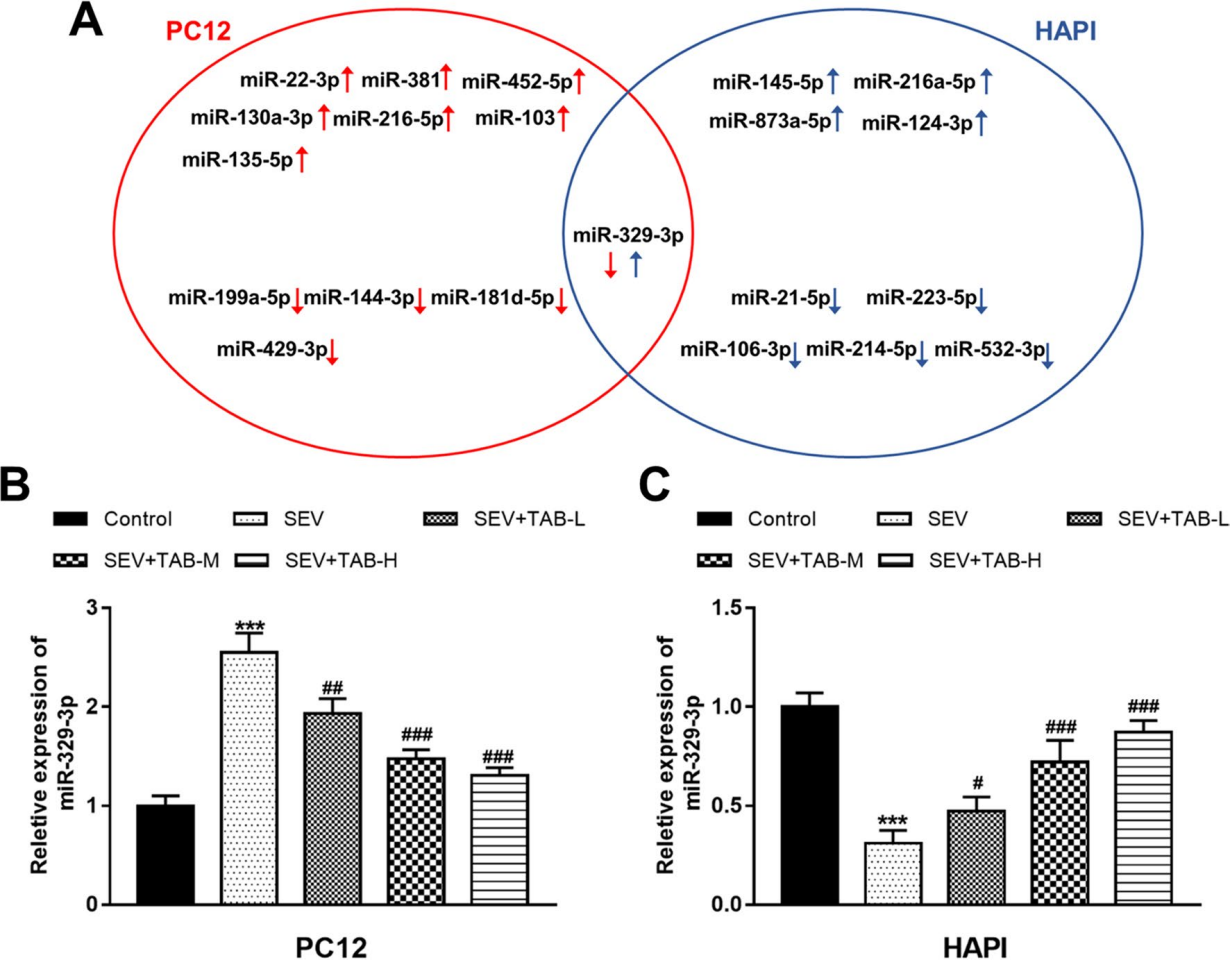
miR-329-3p expression was differentially expressed in PC12 and HAPI cells after TAB treatment. **A** The differentially expressed miRNAs by TAB treatment in PC12 and HAPI received sevoflurane exposure. **B**, **C** miR-329-3p expression was increased in PC12 cells, but was decreased in HAPI by sevoflurane exposure, and these changes caused by sevoflurane were reversed by TAB treatment. ***P < 0.001 compared to control; ^#^*P* < 0.05, ^##^*P* < 0.01, ^###^*P* < 0.001 compared to SEV

This study performed a miRNA microarray assay to screen the differentially expressed miRNAs caused by TAB administration in sevoflurane-treated PC12 and HAPI cells. As show in Table 2; Fig. 4A, 5 miRNAs were found to be upregulated, and 5 miRNAs were downregulated by TAB in PC12 cells. In HAPI cells, TAB significantly upregulated 5 miRNAs and downregulated 5 miRNAs (Table 3; Fig. 4A). Interestingly, in both PC12 and HAPI cells, miR-329-3p was found differentially expressed, but its expression was downregulated in PC12 and upregulated in HAPI (Fig. 4A). According to qRT-PCR, sevoflurane promoted miR-329-3p expression in PC12, while inhibited miR-329-3p in HAPI, and the expression of miR-329-3p was confirmed to be inhibited by TAB in PC12 cells, but was promoted by TAB in HAPI (Fig. 4B, C). In TAB-M and –H groups, the regulatory effects of TAB on miR-329-3p expression were more significant (all *P* < 0.001).

**Table 2:** Differentially expressed miRNAs (fold change > 2 or < 0.5) induced by TAB administration in sevoflurane-treated PC12 cells

miRNAs	TAB–	TAB+	Fold change
Upregulated			
rno–miR–22–3p	0.93	4.51	4.85
rno–miR–381	1.12	4.60	4.11
rno–miR–452–5p	1.25	4.56	3.65
rno–miR–130a–3p	0.55	1.57	2.85
rno–miR–216–5p	0.63	1.74	2.76
rno–miR–103	0.33	0.71	2.16
rno–miR–135a–5p	2.75	5.61	2.04
Downregulated			
rno–miR–199a–5p	1.94	0.66	0.34
rno–miR–329–3p	2.89	1.01	0.35
rno–miR–144–3p	2.56	1.08	0.42
rno–miR–181d–5p	1.85	0.89	0.48
rno–miR–429–3p	1.32	0.65	0.49

**Fig. 5:**
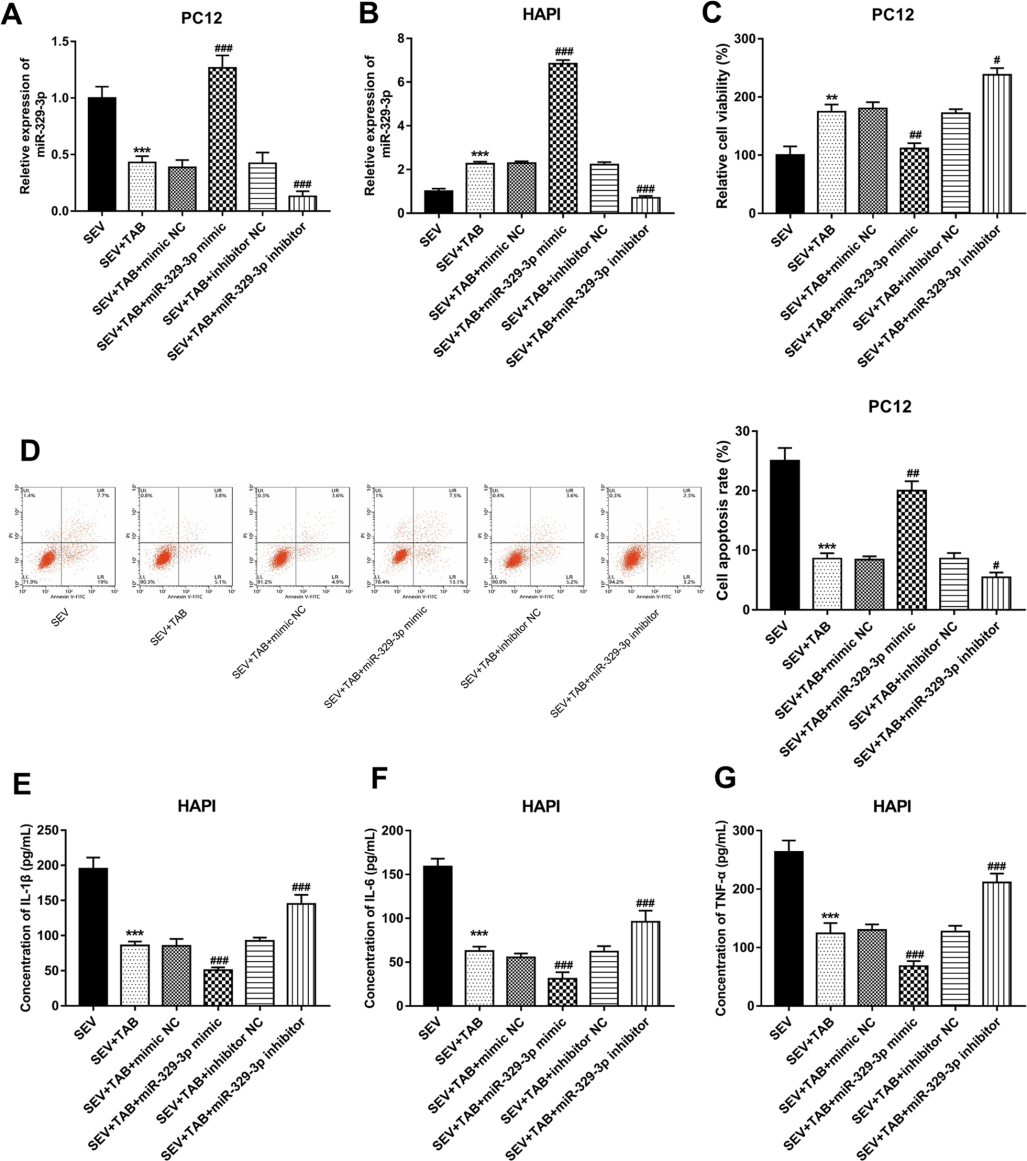
miR-329-3p mediated the effects of TAB on PC12 cell viability and HAPI inflammation. **A**, **B** miR-329-3p was significantly upregulated by the miR-329-3p mimic, but was downregulated by the miR-329-3p inhibitor. **C**, **D** The effects of TAB on PC12 cell viability and apoptosis were reversed by miR-329-3p overexpression, but were enhanced by miR-329-3p inhibition. **E**–**G** The reduced IL-1β (**E**), IL-6 (**F**) and TNF-α **G** induced by TAB were further inhibited by miR-329-3p overexpression, but was elevated by the knockdown of miR-329-3p. **P < 0.01, ***P < 0.001 compared to SEV; ^#^*P* <0.05, ^##^*P* <0.01, ^###^*P *< 0.001 compared to SEV+TAB.

**Table 3:** Differentially expressed miRNAs (fold change > 2 or < 0.5) induced by TAB administration in sevoflurane-treated HAPI cells

miRNAs	TAB–	TAB+	Fold change
Upregulated			
rno–miR–145–5p	0.41	1.62	3.96
rno–miR–216a–5p	0.59	2.02	3.42
rno–miR–329–3p	1.02	3.17	3.11
rno–miR–873a–5p	0.43	0.97	2.25
rno–miR–124–3p	0.71	1.55	2.19
Downregulated			
rno–miR–21–5p	2.22	0.40	0.18
rno–miR–223–5p	1.74	0.45	0.26
rno–miR–106–3p	2.35	0.75	0.32
rno–miR–214–5p	1.34	0.44	0.33
rno–miR–532–3p	1.85	0.76	0.41

### miR-329-3p mediates the effects of TAB on PC12 cell viability and the inflammation of HAPI cells

**Fig. 6:**
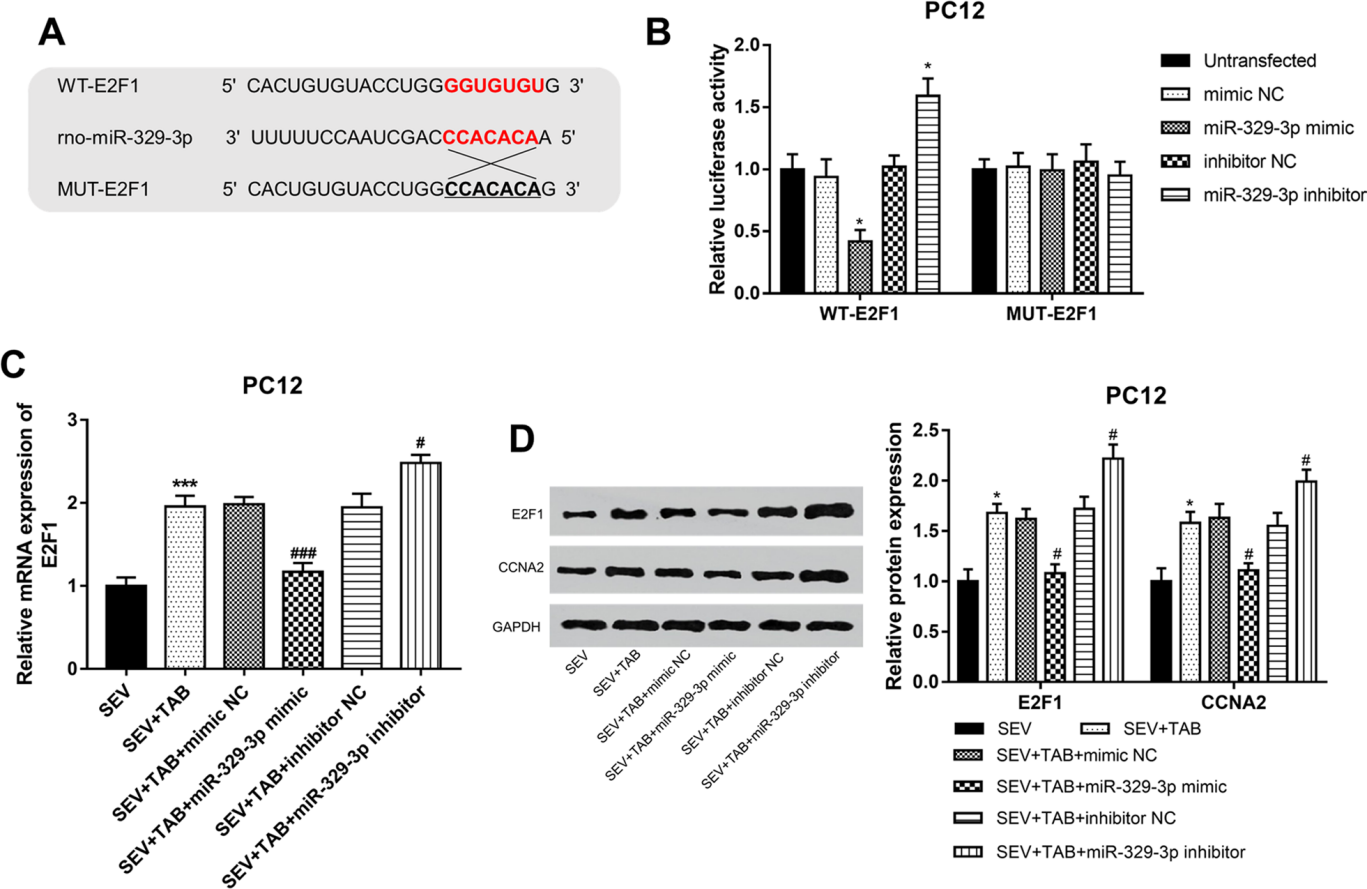
miR-329-3p directly targeted E2F1 and inhibited the E2F1/CCNA2 pathway in PC12 cells. **A** The complementary sequence of miR-329-3p at the 3'-UTR of E2F1. **B** miR-329-3p significantly inhibited the relative luciferase activity in PC12 cells with WT-E2F1 (*P < 0.05). **C** E2F1 mRNA expression was enhanced by TAB in PC12 cells exposed to sevoflurane, and this effect was further enhanced by miR-329-3p inhibition, but was weakened by miR-329-3p overexpression (***P < 0.05 compared to SEV; ^#^*P* <0.05, ^###^*P *< 0.001 compared to SEV+TAB). **D** miR-329-3p overexpression significantly inhibited the protein expression of E2F1 and CCNA2, while miR-329-3p reduction led to the opposite effects on E2F1 and CCNA2 protein expression (*P < 0.05 compared to SEV; ^#^*P* <0.05 compared to SEV+TAB)

The expression of miR-329-3p was significantly upregulated by miR-329-3p mimic, but was downregulated by miR-329-3p inhibitor in both PC12 and HAPI cells (all *P* < 0.001, Fig. 6A, B). For PC12 cell viability and apoptosis, it is found that the overexpression of miR-329-3p weakened TAB-induced increase in cell viability and inhibition in cell apoptosis, while that the knockdown of miR-329-3p enhanced the effects of TAB on cell viability and apoptosis (all *P* < 0.05, Fig. 6C, D). For the inflammatory response in HAPI cells, the decreased levels of IL-1β, IL-6 and TNF-α by TAB were further inhibited by miR-329-3p upregulation, but were reversed by miR-329-3p reduction (all *P* < 0.001, Fig. 6E–G).

### Mir-329-3p participates the neuroprotection of TAB by the E2F1/CCNA2 pathway in PC12 cells

**Fig. 7:**
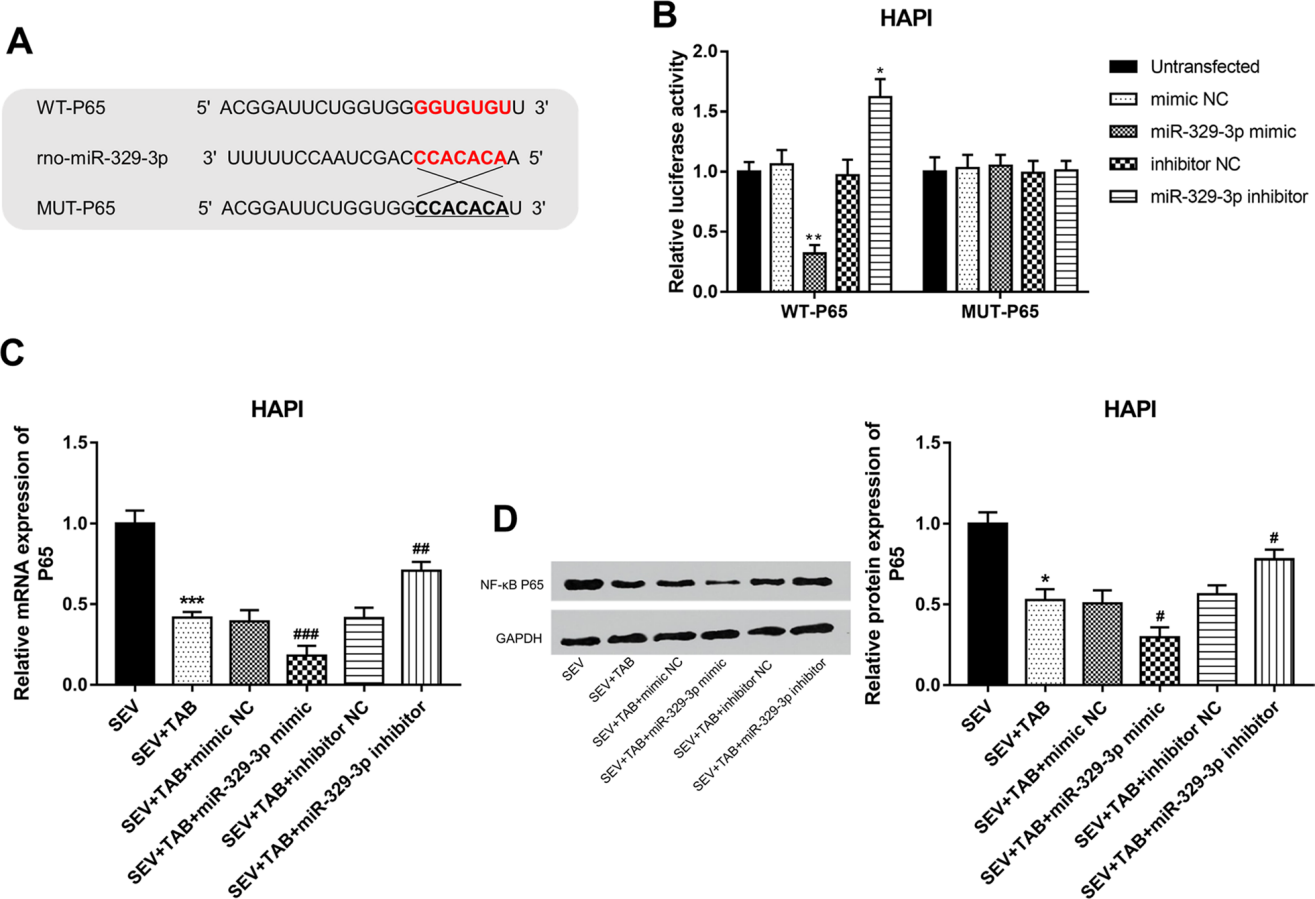
miR-329-3p directly targeted NF-κB P65 and inhibited the NF-κB pathway in HAPI cells. **A** The complementary sequence of miR-329-3p at the 3'-UTR of NF-κB P65. **B** miR-329-3p significantly inhibited the relative luciferase activity in HAPI cells with WT- NF-κB P65 (*P < 0.05). **C** NF-κB P65 mRNA expression was suppressed by TAB in HAPI cells exposed to sevoflurane, and this effect was further enhanced by miR-329-3p mimic, but was weakened by miR-329-3p reduction (***P < 0.05 compared to SEV; ^##^*P* <0.01, ^###^*P *< 0.001 compared to SEV+TAB). **D** miR-329-3p overexpression significantly inhibited the protein expression of NF-κB P65, while miR-329-3p reduction led to the opposite effects on NF-κB P65 protein expression (*P < 0.05 compared to SEV; ^#^*P* <0.05 compared to SEV+TAB).

A complementary sequence of miR-329-3p was found in the sequence of 3’-UTR of E2F1 (Fig. 7A), and the subsequent luciferase reporter assay confirmed that miR-329-3p overexpression significantly inhibited, but miR-329-3p reduction promoted the relative luciferase activity in the group transfected with WT-E2F1 (*P* < 0.05, Fig. 7B). Both the mRNA and protein expression of E2F1 was found to be promoted by TAB (*P* < 0.05), and was further enhanced by miR-329-3p reduction (*P* < 0.05), while the TAB-induced E2F1 upregulation was inhibited by miR-329-3p overexpression (*P* < 0.05) (Fig. 7C, D). Additionally, the downstream protein CCNA2 was also found to be increased by TAB treatment (*P* < 0.05, Fig. 7D), and the effects of miR-329-3p on CCNA2 protein expression were similar to the effects on E2F1.

### miR-329-3p/NF-κB pathway mediates the effects of TAB on inflammation in HAPI cells

The 3’-UTR of NF-κB P65 contained a complementary sequence of miR-329-3p (Fig. 5A), and the luciferase activity of HAPI cells transfected with WT-P65 was significantly inhibited by miR-329-3p upregulation, but was promoted by the miR-329-3p knockdown (*P* < 0.05, Fig. 5B). By checking the mRNA and protein expression of P65, it is found that TAB treatment led to significantly downregulated P65 in HAPI cells compared with the cells treated with only sevoflurane exposure (*P* < 0.05, Fig. 5C, D). As expected, both the mRNA and protein P65 expression was enhanced by miR-329-3p reduction, but was decreased by miR-329-3p overexpression (*P* < 0.05, Fig. 5C, D).

## Discussion

The active ingredients of TCM have attracted a great deal of attention in drug development in various human diseases. This study evaluated the protective effects of TAB against sevoflurane-induced cognitive impairments in rats, and preliminarily explored the underlying mechanisms in neurons and microglia. The findings of the present study demonstrated that TAB could significantly alleviate the cognitive dysfunction in rats inhaled sevoflurane, and the neurotoxicity of PC12 cells and neuroinflammation of HAPI cells induced by sevoflurane were relieved by TAB treatment. In addition, a miRNA microarray assay indicated that miR-329-3p was differentially expressed in PC12 and HAPI cells after TAB administration, and the aberrant miR-329-3p might mediate the neuroprotection of TAB in PC12 and HAPI cells through the E2F1/CCNA2 pathway and the NF-κB signaling.

The biological function of TAB has been previously analyzed and reported. Zhang et al. (2013). In breast cancer cells, TAB was documented to inhibit Taxol resistance by suppressing the expression and activity of P-glycoprotein (Zhang et al. 2014; Shi et al. 2020). Of note, the function of TAB in central nervous system has also received attention. Wang et al. (2019). POCD is a commonly occurred postoperative complication, which has been reported to be closely related with anesthesia and aging (Lin et al. 2020). Sevoflurane is the most widely used inhalation anesthetic, and the cognitive impairments induced by sevoflurane has been considered the major pathomechanism of POCD (Guo et al. 2020). In this study, *in vivo* cognitive dysfunction model was constructed by inhalation of sevoflurane in rats, and the cognitive function, learning and memory abilities of the animals were as expected to be impaired by sevoflurane exposure. In the rats received TAB administration, the behavior of rats did not show changes, and we were surprised to find that the sevoflurane-induced cognitive impairments were significantly alleviated by TAB according to the MWM and FCT tests, and the alleviation was more significant by medium (40 µg/mL) and high (80 µg/mL) concentration TAB. These results provide further evidence for the neuroprotective effects of TAB, and first report the biological function of TAB in sevoflurane-induced cognitive impairments.

According to the previous pathogenesis analysis of sevoflurane-induced cognitive impairments, anesthetic-related neurotoxicity and neuroinflammation are the most important events to aggravate disease progression (Mutch et al. 2018; Safavynia and Goldstein 2018). Chikusetsu saponin IVa has been reported to alleviate sevoflurane-induced cognitive impairments by inhibiting neuronal cell apoptosis and neuroinflammation (Shao et al. 2020). Cistanches has been found to have neuroprotective effects against sevoflurane-induced cognitive dysfunction by regulating inflammatory response in microglia (Peng et al. 2020). These studies documented the important roles of neuronal viability and neuroinflammation played in cognitive impairments induced by sevoflurane. Therefore, differentiated PC12 and HAPI cells were exposed to sevoflurane to obtain the neuron injury cell model and microglia with active inflammation. By the exposure of sevoflurane, PC12 cell viability was inhibited, the cell apoptosis rate of PC12 cells was promoted, and the pro-inflammatory cytokine levels of HAPI cells were increased. And it is gratifying that TAB treatment significantly improved the cell viability and inhibited the cell apoptosis of PC12 cells under sevoflurane exposure conditions, and that the activated inflammation in HAPI cells induced by sevoflurane was markedly inhibited by TAB, which might indicate the potential mechanisms for the neuroprotective effects of TAB against sevoflurane-induced cognitive impairments in rats.

Accumulated studies have showed that miRNAs play critical role in the biological function of TCM nature products. For example, dioscin has cardioprotective effects in doxorubicin-induced cardiotoxicity by regulating miR-140-5p-medicated myocardial oxidative stress (Zhao et al. 2018). Paeonol could increase the survival of sepsis mice by attenuating inflammatory responses through upregulating miR-339-5p (Mei et al. 2019). Ginsenoside Rg1 and Acori graminei Rhizoma have protective roles by inhibiting neuronal cell apoptosis via increasing miR-873-5p expression in Alzheimer’s disease (Shi et al. 2018). Therefore, the present study performed a miRNA microarray assay, and found that miR-329-3p was differentially expressed in both PC12 and HAPI cells after the treatment of TAB. Interestingly, miR-329-3p was found to be expressed negatively between PC12 and HAPI cells, which was downregulated in PC12 but upregulated in HAPI by TAB. In addition, the in vitro analysis results showed that the overexpression of miR-329-3p could reverse the effects of TAB on PC12 cell viability and apoptosis, but enhanced the effects of TAB on the inflammatory responses in HAPI cells. It is well known that miRNAs can target more than one targeted genes, consequently playing different roles in various cell types and pathological processes (Bartel 2009; Hamberg et al. 2016). The differential expression patterns of miR-329-3p induced by TAB between PC12 and HAPI might indicate that miR-329-3p served distinct roles when TAB regulating neuronal viability and neuroinflammation.

In order to find the cause of the opposite expression results of miR-329-3p in PC12 and HAPI cells, and to further explore the molecular mechanisms of TAB, the target genes of miR-324-3p were analyzed. miR-329-3p has been reported to regulate cell viability by targeting E2F1, which is an important regulator of cell cycle and proliferation (Liu et al. 2020a, b). In this study, E2F1 was determined as a target gene of miR-329-3p, and its expression was significantly promoted by miR-329-3p reduction in PC12 cells. In addition, CCNA2, as a key molecule of the E2F1/CCNA2 pathway, was also found to be enhanced by the knockdown of miR-329-3p in PC12 cells. Thus, a conclusion was deduced that miR-329-3p inhibition might mediate the neuroprotective effects of TAB through the E2F1/CCNA2 pathway. Deregulated NF-κB signaling leads to inflammatory responses, which can be detected in the development and progression of various inflammation-related diseases (Zusso et al. 2019; Fan et al. 2013). This study found that NF-κB P65 had a complementary sequence of miR-329-3p, and was a target gene of miR-329-3p. Both the mRNA and protein expression of NF-κB P65 was found to be inhibited by the overexpression of miR-329-3p, and the regulatory effects of TAB on NF-κB signaling pathway were reversed by the inhibition of miR-329-3p, which suggested that miR-329-3p might mediate the anti-inflammation effects of TAB in sevoflurane-induced cognitive impairments through inhibiting the NF-κB signaling.

Although this study first reported the neuroprotective role and potential mechanisms of TAB in sevoflurane-induced cognitive impairments, the mechanisms of TAB remained a preliminary research level. Because of that the model animals needed to be tested for cognitive function in vivo, the hippocampal tissues could not be obtained from rats. Thus, this study did not use primary neuron and microglia, and the mechanisms of TAB explored in this study were not confirmed in rats. The lack of confirmation for the mechanisms in vivo is one of the major limitations of this study, and we will use more animal models to verify the conclusion of this study. Additionally, in the cell experiments, this study used PC12 cells for neuronal apoptosis analysis, and HAPI for neuroinflammation analysis. However, the inflammation changes in PC12 cells, and the cell viability of HAPI cells under TAB administration were not explored. The exploration results of this aspect may provide some interesting to uncover the biological function of TAB, and future studies are necessary to deeply explore the function and mechanisms of TAB.

In conclusion, the findings of this study provide evidence for the protective role of TAB against sevoflurane-induced cognitive impairments by alleviating neurotoxicity and neuroinflammation. miR-329-3p plays an important role in the molecular mechanisms of TAB, which mediates the regulatory effects of TAB on neuronal cell viability and apoptosis through the E2F1/CCNA2 pathway and enhances the anti-inflammation effects of TAB in microglia through the NF-κB signaling. According to our analysis results, TAB maybe a promising TCM nature product for the treatment of POCD induced by inhaled anesthetics.

## Data Availability

The data used to support the findings of this study are available from the corresponding author upon reasonable request.

## Abbreviations

TAB: Trametenolic acid B
POCD: Postoperative cognitive dysfunction
TCM: Traditional Chinese Medicine
miRNAs: MicroRNAs
UTR: Untranslated region
E2F1: E2F transcription factor 1
NF-κB: Nuclear factor-κB
CCNA2: Cyclin A2
DMEM: Dulbecco’s Modified Eagle’s Medium
FBS: Fetal bovine serum
PBS: Phosphate buffered saline
CCK-8: Cell Counting Kit-8
FCT: Fear conditioning test
OFT: Open field test
ELISA: Enzyme-linked immunosorbent assay
(TNF) -α: Tumor necrosis factor
